# Post traumatic intra thoracic spleen presenting with upper GI bleed! – a case report

**DOI:** 10.1186/1471-230X-6-38

**Published:** 2006-11-28

**Authors:** Deepak Hariharan, Rishi Singhal, Sonali Kinra, Andrew Chilton

**Affiliations:** 1 Hepato biliary & pancreatic Surgery, Royal London Hospital, London, UK; 2 General Surgery, University Hospitals Coventry and Warwickshire, Coventry, UK; 3 Medical Gastroenterology, Kettering General Hospital, Kettering, UK

## Abstract

**Background:**

Isolated splenic vein thrombosis with left sided portal hypertension is a rare cause of upper gastrointestinal bleed. Diagnosis is difficult and requires a high index of suspicion, especially in patients presenting with gastrointestinal bleed in the presence of splenomegaly and normal liver function tests.

**Case presentation:**

A 64 year old male presented with haematemesis and melaena. An upper gastrointestinal endoscopy revealed the presence of antral erosions in the stomach and fundal varices. A computerised tomography scan of abdomen confirmed the presence of a diaphragmatic tear and the spleen to be lying in the left hemi thorax. The appearances of the splenic vein on the scan were consistent with thrombosis.

**Conclusion:**

Left sided portal hypertension as a result of isolated splenic vein thrombosis secondary to trauma is rare. The unusual presentation of our case, splenic herniation into the left hemithorax, causing fundal varices leading to upper gastrointestinal bleed 28 years after the penetrating injury, makes this case most interesting. We believe that this has not been reported in literature before.

## Background

Isolated splenic vein thrombosis with left sided portal hypertension is a rare cause of upper gastrointestinal bleed [1]. Diseases of the pancreas have been identified as the most common cause of isolated splenic vein thrombosis [2]. Thrombophilia, myeloproliferative disorders, gastric, renal pathologies, retroperitoneal fibrosis, wandering spleen, lymphomas or sarcomas and iatrogenic causes such as partial gastrectomy, umbilical vein catheterisation, Warren-Zeppa distal splenorenal shunt surgery and splenectomy are the other rare causes associated [2-4].

Diagnosis is difficult and requires a high index of suspicion, especially in patients presenting with gastrointestinal bleed in the presence of splenomegaly and normal liver function tests [4].

## Case presentation

A 64 year old Caucasian male presented to the Emergency Department with haematemesis and melaena for 3 days. He was a known smoker with no history of analgesic or alcohol abuse. 28 years previously, he had sustained a stab injury to left hypochondrium for which he was managed conservatively.

On examination he was pale, tachycardic and hypotensive (pulse rate of 106/min, blood pressure of 94/55 mm of Hg and saturating 100% on air). Systemic examination revealed no abnormality except black tarry stools in the rectum.

**Figure 1:**
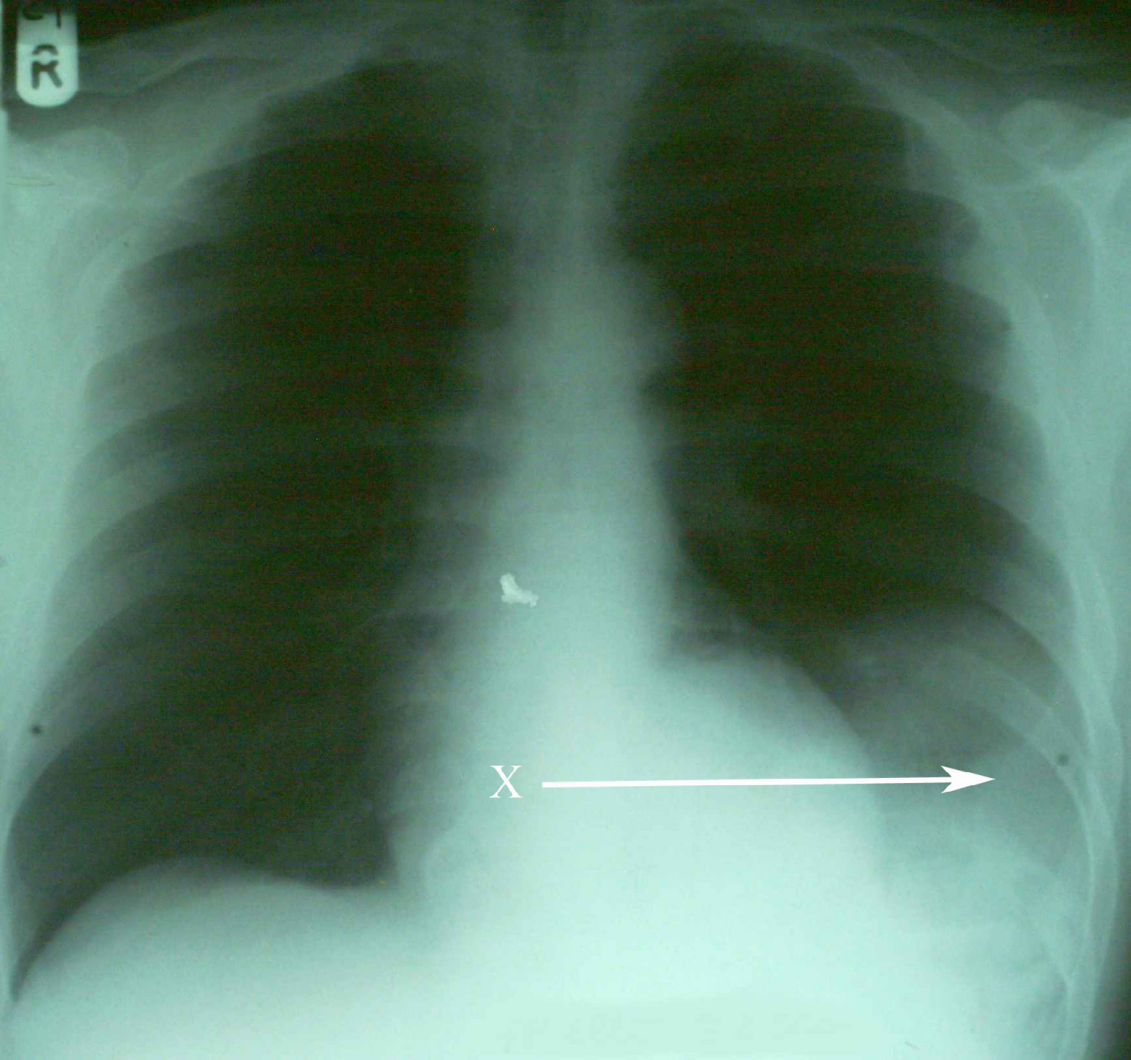
A radiograph of the chest demonstrating an opacity in the left lower zone (X).

Haemoglobin concentration on admission was 5.9 gm/dl, with a serum urea concentration of 16.2 mmol/lit and serum creatinine of 116 mmol/lit. Liver function test including clotting profile, auto antibody and hepatitis B & C screen were normal. A chest radiograph done revealed opacity in the left lower zone (Fig 1).

He responded transiently to fluid resuscitation with the haemoglobin concentration falling to 4.9 gm/dl. An urgent upper gastrointestinal endoscopy was done, which revealed the presence of antral erosions in the stomach and complex gastricfundal varices. No therapeutic intervention was performed.

An ultrasound scan of abdomen revealed an enlarged spleen (15.2 cms), portal vein measuring 11 mm in diameter (upper limit of normal) and prominent veins noted at the splenic hilum. The liver and biliary tree appeared normal.

**Figure 2:**
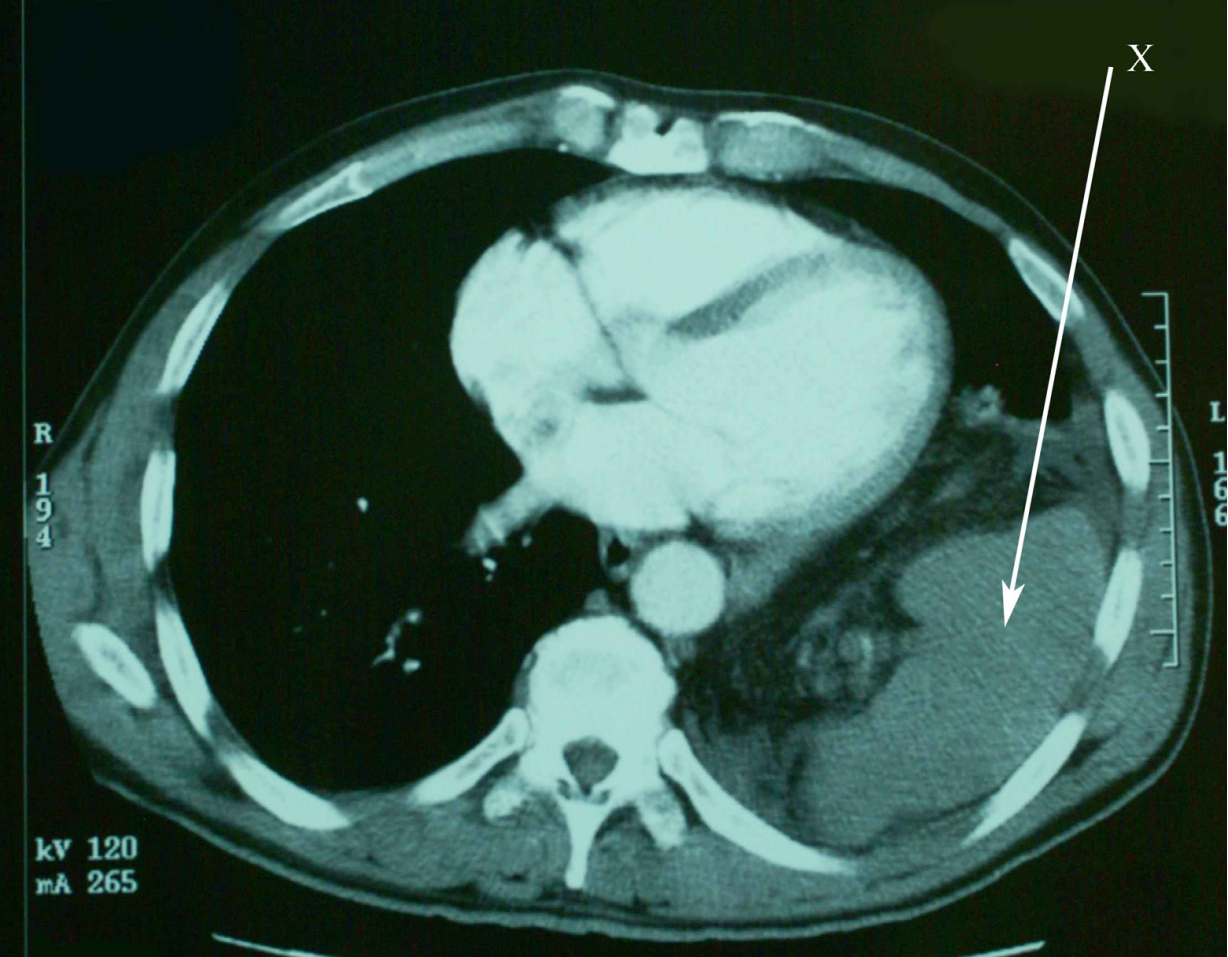
An axial slice from computerised tomography confirming the spleen in the chest (X).

A computerised tomography scan of abdomen in arterial and venous phase confirmed the presence of a diaphragmatic tear and the spleen to be lying in the left hemi thorax (Fig 2). Considerable number of varices was demonstrated in the splenic hilum and perigastric region. The portal vein, its confluence with the splenic vein and the superior mesenteric vein were patent. The appearances of the splenic vein on the scan were consistent with thrombosis.

A diagnosis of left sided portal hypertension as a result of isolated splenic vein thrombosis secondary to trauma causing a diaphragmatic tear and splenic herniation into the left hemi thorax was made. The patient was offered splenectomy with diaphragmatic repair to treat his splenic vein thrombosis and gastric varices.

The patient declined to have surgery and self discharged himself against medical advice.

## Discussion

Isolated splenic vein thrombosis is a rare clinical syndrome that may lead to life threatening haemorrhage from isolated gastric varices. The splenic vein lies inferior to the splenic artery and posterior to the pancreas, hence the aetiology of splenic vein thrombosis is most often related to pancreatic disease [4].

Splenic vein thrombosis after splenectomy for trauma and iatrogenic causes such as partial gastrectomy, umbilical vein catheterisation, Warren-Zeppa shunt have been described in literature [4].

The pathogenesis of splenic vein thrombosis following trauma, is a result of venous stasis, vessel wall dysfunction and alteration in clotting mechanism in the form of hypercoagulability [5]. Immobility following trauma leads to venous stasis, reduced venous return and endothelial cell dysfunction because of decreased oxygen and nutrient delivery [5]. Neutrophil production and activation of cytokines leads to activation of platelets, intrinsic and extrinsic coagulation pathways [6]. Post trauma there is a decrease in circulating levels of plasma anti thrombin III [7,8], protein C/S and plasmin [9]. The net result is of a pro coagulant state.

The longest delayed presentation of traumatic diaphragmatic hernia is 50 years from the time of original injury [10]. There are usually three phases of presentation for traumatic diaphragmatic rupture, namely acute phase, interval phase & obstructive phase [11]. Ball et al in their series observed that those who presented in the acute phase had usually left sided tears and those who had a delayed presentation had significant right sided tears causing herniation [12].

This is in contrast to our case where the penetrating injury was sustained in the left hypochondrium causing splenic herniation and leading him to present with upper gastrointestinal bleed secondary to splenic vein thrombosis 28 years later. We believe that the hypercoagulability was accentuated as a result of the aberrant position of the spleen causing venous stasis and sluggish flow through a kinked splenic vein.

Paramount to establishing the diagnosis of isolated splenic vein thrombosis is a high index of suspicion often aroused by a history of trauma in the vicinity of splenic vein [13].

The symptoms and signs most commonly associated with isolated splenic vein thrombosis is gastrointestinal bleeding and abdominal pain along with splenomegaly and normal liver function tests [14].

A thrombus in the splenic vein causes the venous out flow to return to the portal vein by the way of low pressure collaterals namely the short gastric veins, the veins of the upper half of the stomach, the coronary vein and the gastroepiploic veins [3]. Increased flow across the vessels creates a local form of extrahepatic portal hypertension sometimes referred to as left sided or sinistral portal hypertension [4]. The hypertensive short gastric veins cause increased pressure within the sub mucosal veins of the gastric fundus resulting in gastric varices [15].

It is difficult to diagnose isolated splenic vein thrombosis both endoscopically and radiologically [3]. Gastric varices may not be recognised with endoscopy [3]. Useful investigations are ultrasound examination, along with contrast enhanced CT, upper GI endoscopy and at times digital subtraction angiography [3]. One prospective study recommended the use of endoscopic ultrasonography, a technique quite sensitive for the determination of isolated gastric varices, in patients with high clinical suspicion [3].

Splenectomy is the procedure of choice in the management of haemorrhage due to isolated splenic vein thrombosis [3]. There is no consensus on the treatment of asymptomatic patients, as yet [3].

## Conclusion

Left sided portal hypertension as a result of isolated splenic vein thrombosis secondary to trauma is rare. Diagnosisis difficult and a high index of suspicion is required.

The unusual presentation of our case, splenic herniation into the left hemithorax, causing varices leading to upper gastrointestinal bleed 28 years after the penetrating injury, makes this case most interesting. We believe that this has not been reported in literature before.

## Competing interests

The author(s) declare that they have no competing interests.

## Authors' contributions

DH – literature search, writing

RS – literature search, writing

SK – literature search, writing

AC – Consultant in charge, editing of manuscript

All authors have read and approved the final manuscript.

## Pre-publication history

The pre-publication history for this paper can be accessed here:
